# The impact of flavonoids-rich *Ziziphus jujuba* Mill. Extract on *Staphylococcus aureus* biofilm formation

**DOI:** 10.1186/s12906-020-2833-9

**Published:** 2020-06-17

**Authors:** Weiwei Miao, Lei Sheng, Tao Yang, Guizhen Wu, Minfang Zhang, Juan Sun, Aikemu Ainiwaer

**Affiliations:** 1 Clinical Medical Research Institute, The First Affiliated Hospital of Xinjiang Medical University, Urumqi, 830011 China; 2 Pharmacology Department, Sanquan College of Xinxiang Medical University, Xinxiang, 453000 China; 3 Central Laboratory of Xinjiang Medical University, Urumqi, 830011 China; 4 Heart Center, The First Affiliated Hospital of Xinjiang Medical University, Urumqi, 830011 China

**Keywords:** Flavonoids-rich *Ziziphus jujuba* mill. Extract, *Staphylococcus aureus*, Bacterial biofilm, Crystal violet assays, Confocal laser scanning microscope, Scanning electron microscope

## Abstract

**Background:**

To evaluate the in vitro antibacterial effect of flavonoids-rich *Ziziphus jujuba* Mill. extract (FZM) against the formation of bacterial biofilms (BBFs) in *Staphylococcus aureus*.

**Results:**

FZM can effectively inhibit the formation of *S. aureus* biofilms in vitro. Morphological observation showed a decrease in both biofilm adhesion and thickness. Results of confocal laser scanning microscopy used to detect the thickness of the BBFs showed that FZM treatment reduced the thickness of the BBFs. Furthermore, after the Image-Pro Plus v.6.0 analysis of the fluorescence intensity, FZM treatment reduced the thickness of the BBFs as well as the proportion of green fluorescence. Scanning electron microscopy showed that FZM can disrupt the channels available for substance exchange in the biofilm, thus exposing the bacterial cells and damaging its three-dimensional structures.

**Conclusion:**

FZM can inhibit biofilm formation, improve the bacterial pH environment, and eliminate the hydrophobic effect of reactive oxygen species and flavonoids.

## Background

Jujube (*Ziziphus jujuba* Mill.) fruit, commonly called as the sour or round date, has been used for medical purposes for more than 2000 years. It is an important raw material in food and pharmaceutical industries. Approximately 90% of the world’s *Z. jujuba* is produced in China, where it is widely distributed from Urumqi, Xinjiang. Owing to the extremely strong sedative and tranquilizing effect of this fruit, traditional Chinese medicine often uses the dried seeds for medicinal purposes. The jujube fruit is often wasted, but there are a few studies reporting its medicinal value. The present study analyzed the antibacterial effects of *Z. jujuba* flavonoid on bacterial biofilms [1, 2]. Flavonoids are the major chemical components of jujube [2]. Flavonoids isolated from jujube are reported to exhibit a strong antibacterial effect [3]. Although free individual bacteria are easily cleared by antibiotics, once bacteria form advanced structures, such as bacterial biofilms (BBFs), they are less susceptible to antibiotics, leading to antibiotic resistance [4, 5].

In the present study, we used crystal violet assays, optical microscopy, confocal laser scanning microscopy, and scanning electron microscopy to determine the antimicrobial activity of FZM against *Staphylococcus aureus* biofilm formation.

## Methods

### Materials

*Ziziphus jujuba* Mill. (Purchased from Xinjiang Maidisen Pharmaceutical Company (No.M30062307). The quality inspection report provided by Xinjiang Maidisen Pharmaceutical Company was identified as the dry and mature fruit of *Ziziphus jujuba* Mill. *var.spinosa (Bunge) Hu ex. H.F. Chow* by Miregiuli, the quality inspector of Madison), ethanol anhydrous (Tianjin Yongsheng Chemical Co., Ltd. No.20160303), brain heart infusion broth (BHI; Qingdao Riyong Biological Technology Co., Ltd. No.20160511), crystal violet (Shanghai Hansi Chemical Co., Ltd. No.GC60036), ampicillin (Solarbio No.A8180), normal saline (Sichuan Kelun Pharmaceutical Co., Ltd. No.L116031201), sodium deoxycholate (Sigma–Aldrich, No.30970), 2.5% glutaraldehyde solution (Shanghai Gefan Biological Technology Co., Ltd. No.M011), phosphate buffered solution (PBS; Hyclone, No.SH30256.01), FITC-Concanavalin A (Sigma No.C7642), propidium iodide (PI; Solarbio No.P8080), blood agar base medium (Solarbio, No.RPY-569), cover glass (18 × 18 mm CITO GLAS No.10211818C), slide glass (25 × 75 mm CITO GLAS No.188105), Petri dish (15 × 60 mm, NEST No.705001), 96-well plates (NEST No.20160120801A), CO_2_ incubator (Hera Cell-150 Thermo, Waltham USA), biosafety cabinet (KS-18 Thermo, Waltham USA), microplate reader (Model 680 Bio-Rad, Thermo, Waltham USA), confocal laser scanning microscope (C2 Nikon, Tokyo Japan), electron microscope (CTR6000 Leica, Wetzlar Germany), scanning electron microscope (JSM-6390, Tokyo Japan), *S. aureus* (provided by the Basic Medical College of Xinjiang Medical University, ATCC25923).

### Preparation of *Z. jujube* extract

In this study, dried ripe *Z. jujube* fruit was used. Quality control was conducted by Quality Inspector Mireguli from Xinjiang Madison Pharmaceutical Co., Ltd. Jujubes were picked, cleaned, cored, sliced, dried, and ground into 100-mesh powder. The powder was mixed with ethanol (1:15, g·mL^− 1^) and extracted by reflux for 1.5 h at 70 °C. The supernatant was collected by centrifugation, further concentrated, and dried as jujube extract for further use.

### Impact of FZM on BBF formation

#### Crystal violet assays

An isolated colony of standard *S. aureus* was streaked with an inoculation needle and incubated for 24 h at 37 °C. Subsequently, an isolated colony was diluted in BHI medium and cultivated at 37 °C for 24 h. After vortexing, the bacterial concentration was adjusted to 1 × 10^9^ CFU/mL using McFarland standards.

Biofilms were created by adding 50 μL of the bacterial suspension and 150 μL of the BHI medium containing 1% sucrose to each well of a 96-well plate. They were grown at 37 °C in 5% CO_2_ for 24 h [6]. The supernatants were removed, and the planktonic bacteria were washed away with PBS.

FZM was added to the test group at concentrations of 1.41, 2.81, 5.63, 11.3, 22.5, and 45 μg/mL. Ethanol was used as the negative control, and0.25 mg/mL ampicillin was used as the positive control [7]. There were four replicates of each. The plates were incubated at 37 °C in 5% CO_2_ for 24 h. The supernatants were removed, and the plates were washed three times with PBS. Plates were dried at room temperature for 30 min, after which 200 μL of 0.1% crystal violet (CV) stain was added to each well and left for 15 min, and wells were rinsed with distilled water until transparent. Next, 2% sodium deoxycholate was added to each well to detach the biofilms, and the OD_600nm_ values were measured.

$$ \mathrm{Bacteriostatic}\ \mathrm{rate}\ \left(\%\right)=\left({\mathrm{OD}}_{\mathrm{blank}\ \mathrm{control}}-{\mathrm{OD}}_{\mathrm{test}\ \mathrm{group}}\right)/{\mathrm{OD}}_{\mathrm{blank}\ \mathrm{control}}\times 100\% $$

#### Optical microscopy

Plates were prepared as described above. After 24 h of growth, the 96-well plates were stained with CV and fixed with 2.5% glutaraldehyde. The morphological changes were observed under an optical microscope.

#### Confocal laser scanning microscopy

Sterilized coverslips treated with concentrated sulfuric acid and 75% ethanol were placed in 15 × 60-mm Petri dishes. To each dish, 0.2 mL of the bacterial suspension and 4.8 mL of BHI medium were added. The dishes were incubated for 24 h at 37 °C in 5% CO_2_, and then, the media was discarded. FZM and control treatments were identical to the ones used above. After incubation for 24 h, the coverslips were rinsed with PBS and fixed with 2.5% glutaraldehyde for 30 min. Subsequently, they were rinsed with PBS, stained with FITC-ConA for 30 min, stained with PI for 15 min, and sealed [8]. Finally, they were observed under a confocal microscope with a sub-laser. The centers of the specimen were used to determine the presence of biofilms. The thickest biofilm was imaged, and the thickness was recorded. Bacterial viability was determined using Image-Pro Plus v.6.0 to analyze the intensity of FITC-ConA fluorescence under confocal laser scanning microscopy (CLSM).

#### Scanning electron microscopy

To 15 × 60-mm Petri dishes, 0.2 mL bacterial suspension and 4.8 mL media were added, bacteria were cultured for 24 h at 37 °C in 5% CO_2_, and the media were then discarded. FZM and control treatments were identical to the ones used above. After rinsing with PBS, the dishes were fixed with 2% glutaric acid, dehydrated with anhydrous ethanol, dried with CO_2_, and sputter-coated with gold. They were scanned using scanning electron microscopy (SEM) to determine the presence and morphology of BBFs.

### Statistical analysis

SPSS 20.0 software was used for statistical analysis. If the data were normal, multiple groups were tested by one-way analysis of variance. If the variance was equal, Dunnett’s *t*-test was used for evaluating the difference between each group and the control group. The Dunnett T3 test was used for the variance invariance, where α = 0.05. Data were represented as mean ± standard deviation.

## Results

### Preparation of *Z. jujube* extract

The flavonoid concentration in the jujube extract was 1.44 mg/g at 510 nm.

### Impact of FZM on BBF formation

#### Crystal violet assays

**Fig. 1:**
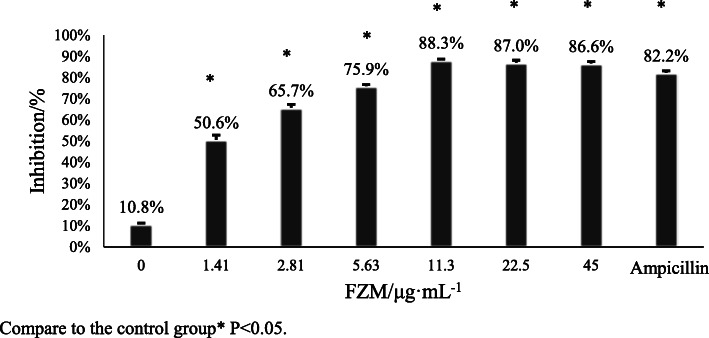
Effect of FZM on biofilm formation evaluated by crystal violet assays cells. Compare to the control group* *P* < 0.05

The presence of FZM decreased the rate of biofilm formation. BBF formation decreased with increased FZM concentration and when the concentration of FZM at 11.3 μg/mL, the inhibitory rate was maximal at 88.3%. After 11.3 μg/mL of FZM, the inhibitory rate tended to be stable. Compared with the control group, these results were significantly different (*p < 0.05*). (Table 1, Fig. 1).

**Table 1:** Effect of FZM on biofilm formation evaluated by crystal violet assays

Group	FZM/μg·mL^− 1^							Ampicillin
	0	1.41	2.81	5.63	11.3	22.5	45	
OD_600_	0.565 ± 0.01	0.313 ± 0.02*	0.217 ± 0.02*	0.177 ± 0.01*	0.074 ± 0.01*	0.082 ± 0.01*	0.085 ± 0.01*	0.113 ± 0.01*
Inhibitory rate%	10.8%	50.6%	65.7%	75.9%	88.3%	87.0%	86.6%	82.2%

Compare to the control group^*^P < 0.05

#### Optical microscopy

**Fig. 2:**
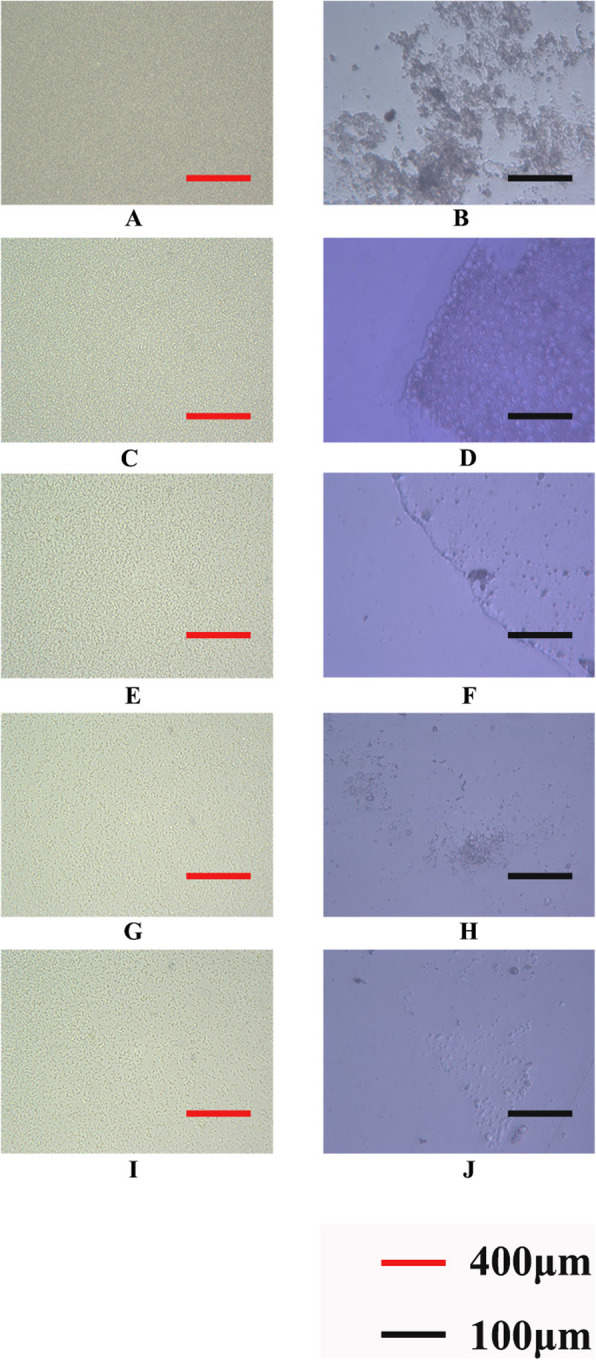
Optical microscope examination. **a**: Control group × 400 μm.**b**: Control group × 100 μm. **c**: 2.81 μg/ml FZM × 400 μm.**d**: 2.81 μg/ml FZM × 100 μm.**e**: 5.63 μg/ml FZM × 400 μm. **f**: 5.63 μg/ml FZM × 100 μm.**g**: 11.3 μg/ml FZM × 400 μm.H:11.3 μg/ml FZM × 100 μm.**i**: Positive group × 400 μm.**j**: Positive group × 100 μm

In the control group, *S. aureus* accumulated and aggregated into clusters with a high bacterial density. The bacteria formed complete biofilms. At 2.81 μg/mL of FZM, The biofilm began to fall off, but there was still mass adhesion, indicating that some of the BBFs had been destroyed. At 5.63 μg/mL of FZM, adhesion was further reduced, and the films were transparent, suggesting that FZM had a significant inhibitory effect on the bacteria at this concentration. At 11.3 μg/mL of FZM, most of the BBFs were detached, and the adhesion area and thickness were smaller than those of the BBFs in the positive control group, indicating that nearly all the biofilms had been removed. In the positive control group, BBF formation was significantly reduced with little adhesion remaining (Fig. 2).

### CLSM

#### BBF thickness and bacterial viability

**Fig. 3:**
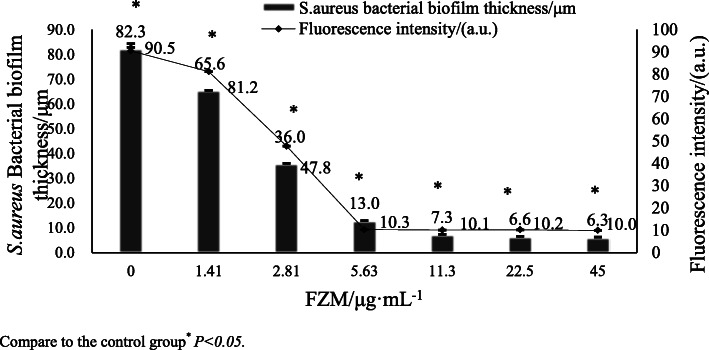
Effect of FZM on biofilm formation evaluated by crystal violet assays cells. Compare to the control group* *P* < 0.05

All concentrations impacted *S. aureus* BBF formation. BBFs thickness decreased with increasing FZM concentration. After 11.3 μg.mL^− 1^ of FZM, the thickness of S.aureus biofilm tends to stabilize The thinnest BBFs were observed at 45 μg/mL of FZM (6.30 ± 0.48 μm). After *S. aureus* BFFs were treated with FZM, the number of viable bacteria significantly decreased. At 11.3 μg/mL of FZM, the intensity of fluorescence significantly decreased. The intensity of bacterial fluorescence was 11.3 μg/mL of FZM (10.1 ± 0.03 a.u.). After 5.625 μg.mL^− 1^ of FZM, the level of fluorescence was stable. The results were significantly different from those of the control group (*p < 0.05*) (Table 2, Fig. 3).

**Table 2:** BBFs thickness and the vital bacterial percentage

Group	FZM/μg·mL^− 1^							Ampicillin
	0	1.41	2.81	5.63	11.3	22.5	45	
Bacterial biofilm thickness/μm	82.3 ± 0.63^*^	65.6 ± 1.05^*^	36.0 ± 0.94^*^	13.0 ± 0.49^*^	7.30 ± 0.75^*^	6.60 ± 0.10^*^	6.30 ± 0.48^*^	2.10 ± 0.48^*^
Fluorescence intensity/(a.u.)	90.5 ± 3.30^*^	81.2 ± 0.52^*^	47.8 ± 0.12^*^	10.3 ± 0.50^*^	10.1 ± 0.03^*^	10.2 ± 0.11^*^	10.0 ± 0.59^*^	16.6 ± 0.43^*^

Compare to the control group^*^P < 0.05

### CLSM

**Fig. 4:**
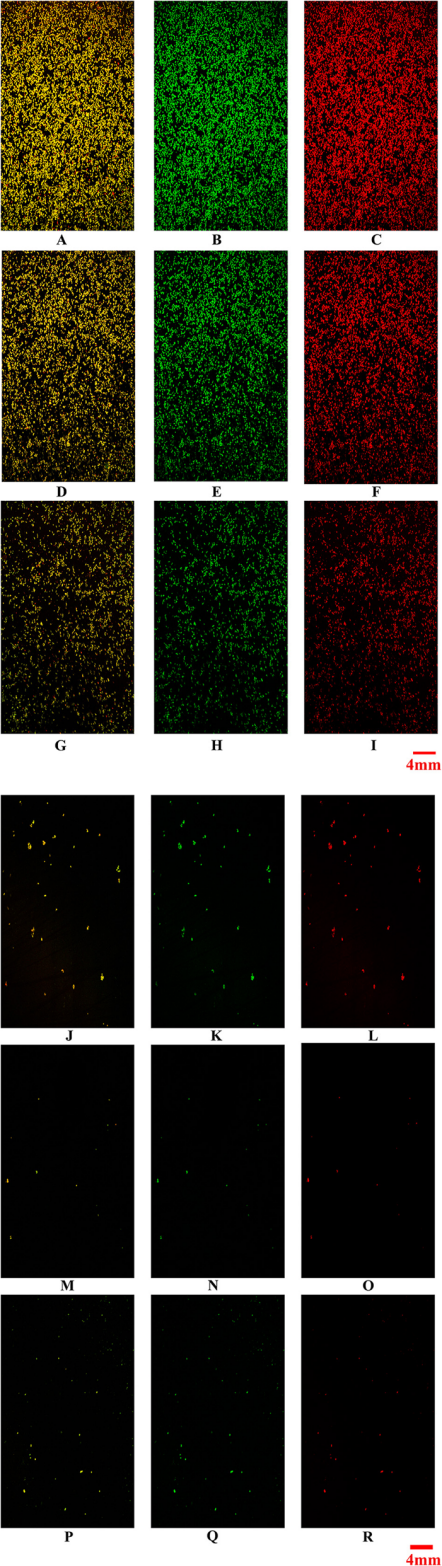
Effect of FZM on biofilm formation evaluated by crystal violet assays cells. **a**:Control group × 4 mm Channels merged. **b**:Control group × 4 mm Green channel. **c**:Control group × 4 mm Red channel. **d**:1.41 μg/ml × 4 mm Channels merged. **e**1.41 μg/ml × 4 mm Green channel. **f**:1.41 μg/ml × 4 mm Red channel. **g**:2.81 μg/ml × 4 mm Channels merged. **h**:2.81 μg/ml × 4 mm Green channel. **i**:2.81 μg/ml × 4 mm Red channel. **j**:5.63 μg/ml × 4 mm Channels merged. **k**:5.63 μg/ml × 4 mm Green channel. **l**:5.63 μg/ml × 4 mm Red channel. **m**:11.3 μg/ml × 4 mm Channels merged. **n**:11.3 μg/ml × 4 mm Green channel. **o**:11.3 μg/ml × 4 mm Red channel. **p**:Positive group × 4 mm Channels merged. **q**:Positive group × 4 mm Green channel. **r**:Positive group × 4 mm Red channel

In the control group, *S. aureus* aggregated in clusters and the overlapping parts of the live and dead bacterial cells appeared yellow (green + red) [9]. The bacteria were embedded in a matrix formed by a number of polysaccharides (green), which exhibited a compact structure with complete biofilm formation. There was less green fluorescence in the cells treated with 1.41 μg/mL of FZM, indicating a reduced BBF polysaccharide matrix. The bacterial distribution was sparse, so BBFs were destroyed from this concentration onwards. At 2.81 μg/mL of FZM, the bacterial density was significantly reduced, although clearly observable bacterial aggregations remained. This suggests that there is enhanced inhibition of FZM at this concentration with some remaining bacterial adhesions. At 5.63 μg/mL of FZM, the fluorescence was weak, most biofilms had disappeared, and the surrounding scattered star-shaped green fluorescence suggested the presence of a few planktonic bacteria. At 11.3 μg/mL of FZM, the fluorescent spots were barely visible, indicating that the biofilms had disappeared at this concentration. In the positive control group, there were a few clusters of low-density bacteria with large shedding of BBFs and only a small number of green and yellow dots. It shows that BBFs has been removed the BBFs had been removed. (Fig. 4-1, Fig. 4-2).

### SEM

**Fig. 5:**
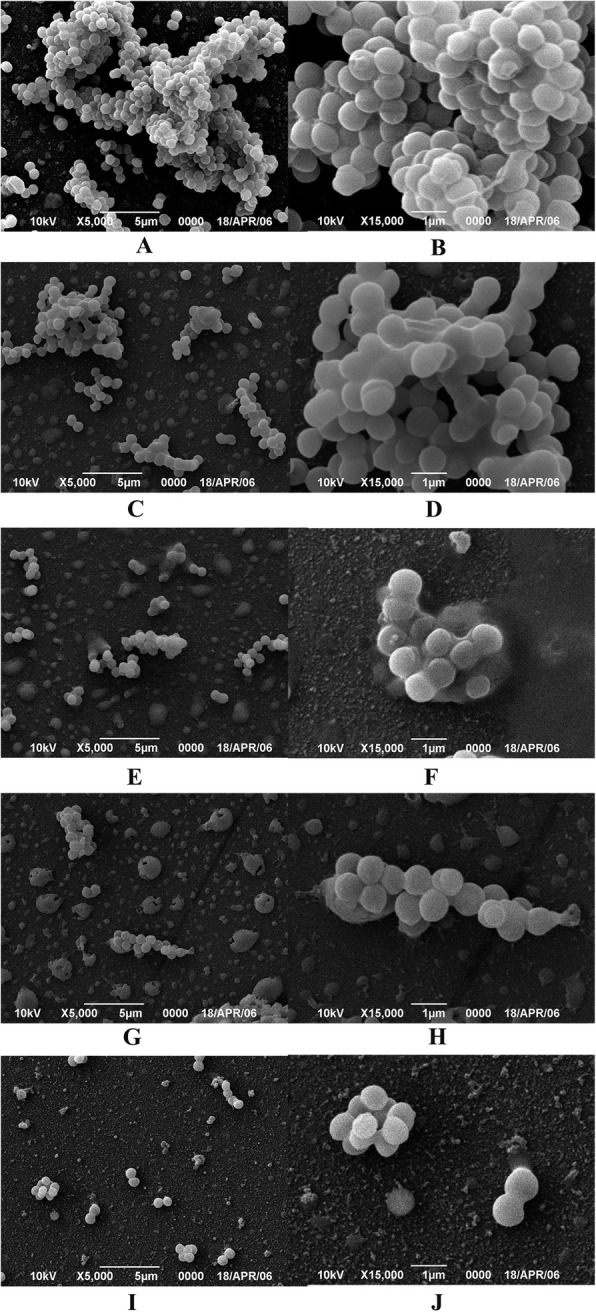
Confocal laser scanning microscope examination. **a**: Control group × 5 μm. **b**:Control group × 1 μm. **c**:2.81 μg/ml × 5 μm. **d**:2.81 μg/ml × 1 μm. **e**:5.63 μg/ml × 5 μm. **f**:5.63 μg/ml × 1 μm. **g**:11.3 μg/ml × 5 μm. **h**:11.3 μg/ml × 1 μm. **i**:Positive group × 5 μm. **j**:Positive group × 1 μm

Different from biofilms formed by other bacteria, S.aureus formed biofilms were more dense, and *S. aureus* revealed grape-like clusters in biofilms [10, 11]. Bacterial mucus filaments were easily identified in the negative control group. These filaments were tightly wrapped in mucus-like substances forming a large and dense membrane-like structure with an uneven appearance. The structure contained channels for water and nutrient exchange, which provided the necessary environment for a low level of bacterial growth and metabolism in the BBF dormant state [12]. The *Staphylococcus aureus* is tightly wrapped on the surface of Petri dish by bacterial biofilm. At 2.81 μg/mL of FZM, large accumulations of *S. aureus* cells and extracellular mucilage were clearly observed, indicating that there was still biofilm adhesion despite the inhibitory effect of FZM. At 5.63 μg/mL of FZM, visible microvilli were observed around the BBFs, and BBFs transmittance was stronger than that in the negative control group, indicating that FZM had some effects on BBFs at this concentration. At 11.3 μg/mL of FZM, most of the cells shrank and contained broken three-dimensional (3D) structures. Few cells were intact, but these were not coated with mucous and were clear, indicating that this concentration had a strong scavenging effect and that the bacterial structure began to disassemble. The density of *S. aureus* was significantly lower in the presence of ampicillin (Fig. 5-1, Fig. 5-2). The bacteria were scattered with clear boundaries and no mucinous coating, indicating a serious loss of BBF formation.

## Discussion

*S. aureus* is an important nosocomial pathogen in hospitals, and it commonly forms biofilms [13]. The current treatments are antibiotics; however, long-term use of antibiotics results in increased resistance. We have shown that reducing the number of bacteria does not reduce biofilm formation [14]. This suggests that antibacterial drugs may not be effective in removing bacterial biofilms. The extracellular polysaccharide coating on biofilms helps bacteria within the membrane to evade the immune system of the human body [15–17]. Studies have shown that at least 65% of human infections are caused by bacteria and biofilm formation [18–21]. Therefore, it is important to investigate effective drugs that remove bacterial biofilms in resistant strains [22].

Natural active ingredients extracted from plants have unique advantages for the treatment of *S. aureus* biofilm infections [14, 23–31]. Flavonoids, such as quercetin, naringenin, and dihydrochalcone, in these extracts have been reported to have antibiofilm effects [23–25, 32]. In addition, kaempferol (flavonoids) can inhibit the biofilm formation of *Staphylococcus aureus* [33]. The mechanism may be due to the fact that the outer membrane of bacteria has a narrow pore protein that slows down the penetration of hydrophilic antibiotics. All flavonoids are hydrophobic, suggesting an increased potential for biofilm treatment [32].

In the present study, the results of the microplate assays indicate that FZM effectively inhibits the in vitro formation of *S. aureus* biofilms. By increasing the concentration of FZM, the rate of biofilm formation reduced steadily. Biofilm thickness is one of the criteria used to assess the ability of compounds to remove biofilms [34]. In our study, we found that FZM reduced the thickness of the biofilms and destroyed the 3D structure, thereby inhibiting biofilm maturation. At the same time, the viable and dead bacteria in the biofilm were significantly reduced, suggesting that FZM can penetrate the biofilm matrix and act directly on the bacteria.

Current research shows that the removal of adhesion proteins, the increase in bacterial pH, and the destruction of the bacterial quorum sensing system are the key factors for removing *S. aureus* biofilms [24, 25, 27, 35]. Studies have confirmed that scavenging free radicals and reactive oxygen species from flavonoids can effectively change the bacterial pH environment, suggesting that this is one of the mechanisms by which flavonoids can remove biofilms. FZM removes reactive oxygen species, has antioxidative and antiinflammatory effects, and improves the intestinal microenvironment [32, 36–44]. In our study, subtle changes in BBFs were observed by SEM. In the negative control, the pores for substance exchange and the 3D structure of the *S. aureus* biofilm were gradually destroyed after FZM treatment. Exopolysaccharides were cleared, and the bacteria were gradually exposed. This further confirmed that FZM can significantly improve BBF detachment. It may remove bacterial exopolysaccharides and disrupt the channels available for substance exchange in the biofilm.

## Conclusion

FZM can inhibit biofilm formation, improve the bacterial pH environment, and eliminate the hydrophobic effect of reactive oxygen species and flavonoids. Further studies are required to investigate the mechanism of action of FZM on the bacterial quorum sensing system.

## Data Availability

The datasets used and/or analyzed during the current study are available from the corresponding author on reasonable request.

## Abbreviations

BBFs: Bacterial biofilms
BHI: Brain heart infusion broth
CLSM: Confocal laser scanning microscope
FZM: Flavonoids-rich *Ziziphus jujuba* Mill. extract
PBS: Phosphate buffered solution
SEM: Scanning electron microscope
